# Expansion of CD4^+^CD25^+^ and CD25^-^ T-Bet, GATA-3, Foxp3 and RORγt Cells in Allergic Inflammation, Local Lung Distribution and Chemokine Gene Expression

**DOI:** 10.1371/journal.pone.0019889

**Published:** 2011-05-19

**Authors:** You Lu, Carina Malmhäll, Margareta Sjöstrand, Madeleine Rådinger, Serena E. O'Neil, Jan Lötvall, Apostolos Bossios

**Affiliations:** Department of Internal Medicine, Krefting Research Centre, Institute of Medicine, The Sahlgrenska Academy, University of Gothenburg, Gothenburg, Sweden; University of Pittsburgh, United States of America

## Abstract

Allergic asthma is associated with airway eosinophilia, which is regulated by different T-effector cells. T cells express transcription factors T-bet, GATA-3, RORγt and Foxp3, representing Th1, Th2, Th17 and Treg cells respectively. No study has directly determined the relative presence of each of these T cell subsets concomitantly in a model of allergic airway inflammation. In this study we determined the degree of expansion of these T cell subsets, in the lungs of allergen challenged mice. Cell proliferation was determined by incorporation of 5-bromo-2′-deoxyuridine (BrdU) together with 7-aminoactnomycin (7-AAD). The immunohistochemical localisation of T cells in the lung microenvironments was also quantified. Local expression of cytokines, chemokines and receptor genes was measured using real-time RT-PCR array analysis in tissue sections isolated by laser microdissection and pressure catapulting technology. Allergen exposure increased the numbers of T-bet^+^, GATA-3^+^, RORγt^+^ and Foxp3^+^ cells in CD4^+^CD25^+^ and CD4^+^CD25^-^ T cells, with the greatest expansion of GATA-3^+^ cells. The majority of CD4^+^CD25^+^ T-bet^+^, GATA-3^+^, RORγt^+^ and Foxp3^+^ cells had incorporated BrdU and underwent proliferation during allergen exposure. Allergen exposure led to the accumulation of T-bet^+^, GATA-3^+^ and Foxp3^+^ cells in peribronchial and alveolar tissue, GATA-3^+^ and Foxp3^+^ cells in perivascular tissue, and RORγt^+^ cells in alveolar tissue. A total of 28 cytokines, chemokines and receptor genes were altered more than 3 fold upon allergen exposure, with expression of half of the genes claimed in all three microenvironments. Our study shows that allergen exposure affects all T effector cells in lung, with a dominant of Th2 cells, but with different local cell distribution, probably due to a distinguished local inflammatory milieu.

## Introduction

Asthma is one of the most common chronic diseases worldwide, with an estimated total prevalence of 5% [1]. Eosinophilic inflammation is a common characteristic linked closely to allergic asthma. Beyond eosinophils, different T cells are important regulators of the inflammatory process in asthma. Indeed, the lung has an extensive network of antigen-presenting cells that provide antigen presentation to T cells in lung-associated lymphoid tissue [2].

CD4^+^ T cells are essential regulators of the immune response and inflammatory diseases. After encountering specific antigen, they become activated, expand their populations and differentiate into various effector T cell subsets, such as T helper type 1 (Th1), Th2, interleukin 17 (IL-17)-producing T helper (Th17) and regulatory T cells (Treg cells). The development of these cells is dictated by their specific transcription factors T-bet, GATA-3, RORγt and Foxp3, respectively [3]–[6]. These cells have been suggested to regulate different aspects of allergic inflammation. For example, the development of allergy and the presence of eosinophilic inflammation are suggested to be driven by a Th2 over-activation, in relation to a reduced Th1 activity [7]. Beyond these two cell types, Th17 and Treg cells have been proposed to interact with other T cells, and interrelate with each other, to determine which type of inflammation is induced [8], [9]. However, no study has directly determined the relative presence, tissue distribution or surrounding inflammatory milieu of each of these T cell subsets concomitantly in a model of allergic airway inflammation.

The aim of the current study was to determine the degree of expansion of different T cell subsets expressing transcription factors for Th1, Th2, Th17 and Treg cells, in the lungs of sensitised mice exposed to allergen. To establish in which microenvironment these cells reside, immunohistochemistry for each transcription factor was performed. General lung inflammation was estimated by measuring the concentration of cytokines released in the lung. The local inflammatory response in the microenvironments where different cells were located was estimated by measuring the expression of 60 inflammatory cytokine, chemokine and receptor genes using real-time RT-PCR array in tissue isolated by laser microdissection and pressure catapulting technology.

## Materials and Methods

### Animals

This study was approved by the Animal Ethics Committee in Gothenburg, Sweden. The permit number is Dnr442–2008 data 2008-12-12. Male C57BL/6 mice, 5 to 6 weeks old, were purchased from Taconic (Ry, Denmark). All mice were kept under standard animal housing conditions and provided with food and water *ad libitum*.

### Allergen sensitisation and allergen exposure

**Figure 1:**
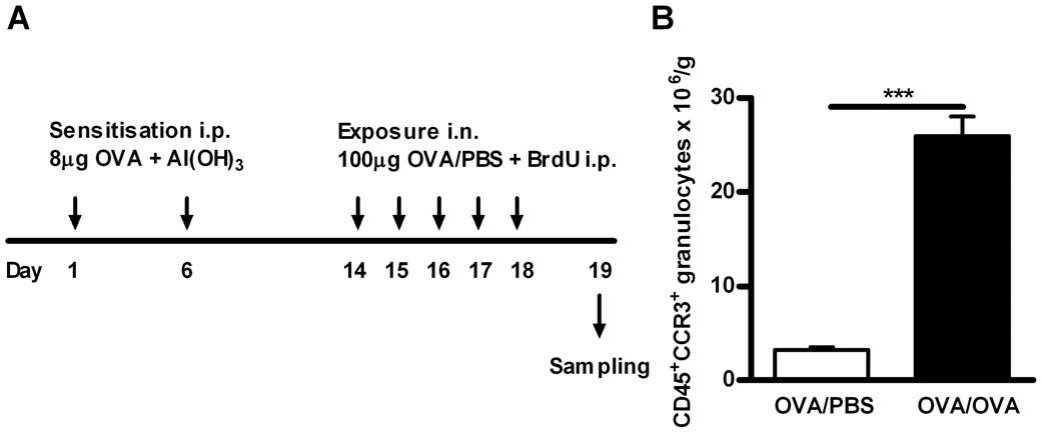
Establishment of allergic airway inflammation. A) Schedule of OVA sensitisation (i.p.), OVA exposure (25 µl intranasal instillation: i.n.), BrdU administration (1 mg i.p.) and tissue sampling in this model of allergic airway inflammation. All animals were sensitised to allergen, but control animals were exposed to PBS instead of OVA. B) Number of lung tissue CD45^+^ CCR3^+^ granulocytes (i.e. eosinophils) per gram of tissue after OVA/PBS or OVA/OVA exposure, Data is shown as mean ± SEM, n  =  5–8 mice/group, ***, *p*<0.001.

Mice were sensitised twice, with an interval of five days, by the intraperitoneal (i.p.) injection of 0.5 ml of 8 µg chicken ovalbumin (OVA)(Sigma-Aldrich®, St Louis, MO, USA) bound to 4 mg aluminium hydroxide (Sigma-Aldrich®) in phosphate buffered saline (PBS). Eight days after the second sensitisation, the mice were divided in two groups. Both groups were briefly anesthetised using isoflurane (Baxter, Deerfield, IL, USA). The exposure group received an intranasal (i.n.) administration of 100 µg OVA in 25 µl of PBS on five consecutive days, while the control group received only PBS (Fig. 1A).

### Evaluation of newly produced inflammatory cells

BrdU (5-bromo-2′-deoxyuridine) is a thymidine analogue that is incorporated into the DNA during the S-phase of the cell cycle by replacing thymidine. All mice were given 4 mg of BrdU (BrdU Flow Kits, BD Pharmingen™, San Diego, CA) to label newly produced cells. BrdU was given at a dose of 0.8 mg in 0.2 ml of PBS by i.p. injection once a day just after allergen exposure, on days 1 to 5 of allergen exposure.

### Sample collection and processing

Samples were collected 24 hrs after the final OVA exposure. The animals were deeply anesthetised with a mixture of xylazin (130 mg/kg, Rompun®, Bayer, Germany) and ketamine (670 mg/kg, Ketalar®, Parke-Davis, England). Firstly, animals were sacrificed by puncturing the right heart ventricle and blood was collected. Secondly, the mice were tracheotomised and bronchoalveolar lavage (BAL) was performed by instilling 0.25 ml PBS through the tracheal cannula, followed by gentle aspiration and a second lavage with 0.20 ml PBS. An additional 1 ml of PBS was used to wash away airway lumen inflammatory cells, before the lungs were perfused and removed to harvest parenchymal inflammatory lung cells. The left lobe was filled with 1 ml of Tissue-Tek® (Sakura Finetek Europe B.V., 2382 AT, Zoeterwoude, NL) and PBS containing 20% sucrose (Sigma-Aldrich®) and immediately frozen in liquid nitrogen. The apical lobe was stored at −80°C and another three lobes, without any connective tissue, were stored on ice in Hanks balanced salt solution (HBSS) (Sigma-Aldrich®) before use.

### Preparation of lung single-cell suspensions

The right lung lobes were weighed and rinsed in a Petri dish before being transferred to a gentleMACS™ C Tube (Miltenyi Biotec GmbH, Bergisch Gladbach, Germany) containing 5.0 ml of HBSS supplemented with 10% fatal calf serum (FCS) (Sigma-Aldrich®), 100 µl Collagenase D solution (final concentration 2 mg/ml) and 20 µl DNase I solution (final concentration 80 U/ml). The mouse lung was dissociated using a gentleMACS Dissociator (Miltenyi Biotec) according to the manufacturer's instructions. Lung cell pellets were collected, washed in PBS supplemented with 10% FCS and the cells saved for FACS analysis. The total number of cells was determined using standard haematological procedures.

### Flow cytometry and gating strategy

#### Flow cytometric analysis of viable leucocytes, eosinophils and T cells

Cells were pre-treated with 2% mouse serum (DAKO) for 15 min to prevent unspecific binding and thereafter stained with the following antibodies; Fluorescein (FITC) labelled anti-CD45 (clone 30-F11, BD Biosciences), Peridinin Chlorophyll Protein Complex (PerCP) labelled anti-CD3e (clone 145-2C11), Phycoerythrin (PE) labelled anti-CD4 (clone H129.19, BD Biosciences) and 7-aminoactinomycin (7-AAD) (BrdU Flow Kits, BD Pharmingen™, San Diego, CA) or a matching isotype control antibody. The cells were incubated for 30 min at 4°C with the antibodies or isotype controls, followed by two washes with washing buffer (PBS +10% FCS) and fixed with Fix/Perm solution (Foxp3 Staining Buffer Set, eBioscience™) overnight at 4°C. The remainder of the protocol is as described for the intracellular staining below, with the exception of the antibody addition. Gating was first set on intact cells based on forward and side scatter characteristics. Viable leucocytes were identified as 7-AAD^-^CD45^+^ cells in a gated population based on forward and side scatter profiles. Mature eosinophils were identified as CD45^+^CCR3^+^SSC^high^; T lymphocytes were identified as CD45^+^CD3^+^ cells; CD4^+^ T cells were identified as CD45^+^CD3^+^CD4^+^ cells.

#### Flow cytometric cell cycle analysis of newly produced and proliferating T helper cell subsets

Lung cells were collected as described above for the staining of different T helper cells, Th1 cells (CD4^+^CD25^+^T-bet^+^), Th2 cells (CD4^+^CD25^+^GATA-3^+^), Th17 cells (CD4^+^CD25^+^RORγt^+^) and Treg cells (CD4^+^CD25^+^Foxp3^+^). Cells were stained with following antibodies; FITC labelled anti-CD4 (clone RM4-5, BD Biosciences) and Alexa Fluor® 700 labelled anti-CD25 (clone PC61, BioLegend, San Diego, CA) or a matching isotype control antibody. Cells were fixed and intracellular staining performed according to the manufacturer's protocol for the Foxp3 staining buffer set (eBioscience™). Intracellular antibodies used included PE labelled anti-T-bet (clone eBio4B10, eBioscience™), PE labelled anti-GATA-3 (clone TWAJ, eBioscience™), PE labelled anti-RORγt (clone AFKJS-9, eBioscience™) and PE labelled anti-Foxp3 (clone FJK-16s, eBioscience™). The subsequent intracellular staining for BrdU and 7-AAD was performed using BrdU Flow Kits (BD Pharmingen™, San Diego, CA) according to the manufacturer's instructions. BrdU was used to identify the newly produced cells and 7-AAD was used as a DNA dye to identify the proliferation of newly produced cells *in situ*. All flow cytometry analyses were carried out using a BD FACSAria™ Flow Cytometer (BD Biosciences, San Jose, CA) with 150 000 events being acquired per sample and analysed with FlowJo Software® (Tri star Inc, Ashland, OR, USA). Due to the cell size and characteristic profile, the non-granulocytes (mononuclear cells) were gated under the intact cells, as described above. CD4^+^ cells were gated based on forward and side scatter characteristics and isotype control.

### Lung histology

The left lobe of the lung was embedded in Tissue-Tek® O.C.T. Compound (Miles, Inc., Elkhart, IN, USA), snap-frozen and stored at −80°C until sliced into 5 µm sections using a cryostat Leica CM1900 UV-cryostat® (Leica Microsystems Nussloch GmbH, Germany).

### Eosinophils

Eosinophils were identified by immunostaining of the major basic protein (MBP), an eosinophil granule protein expressed early in eosinophil-lineage committed cells, as well as in mature eosinophils. (Kind gift from Dr James Lee, Mayo Clinic, Scottsdale, AZ, USA). The immunostaining was performed as described previously [10].

### Th1 and Th2 lymphocytes

Tissue sections were fixed for 30 min in 2% formaldehyde, rinsed in Tris-buffered saline (TBS) and subjected to heat-induced antigen retrieval using Target Retrieval solution, pH 6 (DakoCytomation, Glostrup, Denmark). Endogenous peroxidase was blocked with pre-heated (37°C for 30 min) glucose oxidase solution (PBS supplemented with 0.0064% sodium azide, 0.18% glucose, 0.1% saponin and 1.55 units of glucose oxidase/ml PBS)(all from Sigma) by incubation for 30 min. All washes between the steps were performed using TBS, with or without Tween 20.

For T-bet staining, the blocking of unspecific binding sites was performed by incubating the slides with Normal Horse Serum (ImmPRESS™ REAGENT, Vector Laboratories, Inc., Burlingame, CA). Primary antibodies for the detection of T-bet (rabbit anti-mouse T-bet, clone H-210, Santa Cruz Biotechnology Inc.) were applied, followed by a secondary antibody-anti-rabbit Ig-(ImmPRESS™ REAGENT, Vector Laboratories, Inc). Bound antibodies were visualised by 3.3′-diaminobenzidine (DAB) (DAKO Liquid DAB Substrate-Chromogen System, DakoCytomation) and counterstained with Mayer's Hematoxylin (Sigma). A matched isotype control was used at the same protein concentration as the primary antibody.

For GATA-3 staining, the endogenous biotin was blocked by using the Avidin/Biotin Blocking Kit (Vector Laboratories, Inc.). The biotinylation of the primary antibody, GATA-3, (Mouse anti-GATA-3, clone L50–823, BD Pharmingen™) was prepared by the addition of the antibody to the biotin reagent (DakoCytomation ARK™ (Animal Research Kit)) which was incubated for 15 min, followed by the addition of the blocking reagent. Sections were incubated in the prepared biotinylated antibody mixture for 20 min, followed by horse radish peroxidise (HRP) conjugated streptavidin. The detection of the antibody staining and subsequent counterstaining was performed as above for T-bet.

### T regulatory lymphocytes

For Foxp3 staining, the sections were subjected to heat-induced antigen retrieval using Antigen Retrieval Reagent Basic (R&D Systems Inc., Abingdon, UK) for 30 min. The blocking of endogenous peroxidase was performed as described above, while the blocking of unspecific binding sites and endogenous biotin was performed by incubating the slides with 10% Normal Donkey Serum (Jackson ImmunoResearch Laboratiories. Inc., PA, USA) followed by the use of the Avidin/Biotin Blocking Kit. The primary antibody for the detection of Foxp3 (rat anti-mouse/rat FOXP3, clone FJK-16s, eBiosciences™) was applied, followed by the secondary antibody, biotin-conjugated F(ab')_2_ donkey anti-rat IgG (Jackson ImmunoResearch Laboratories. Inc.) and ExtrAvidin-HRP (Sigma). Antibody detection and counterstaining was performed as above.

### Th17 lymphocytes

For RORγt staining, tissue sections were fixed with 2% formaldehyde for 10 min, rinsed in PBS and incubated with 10% Normal Rabbit Serum (Jackson ImmunoResearch Laboratories, Inc.), followed by incubation with rat anti-mouse/human RORγ (t) (ROR gamma, Retinoid-Related Orphan Receptor gamma) (clone AFKJS-9, eBioscience™). Unless otherwise stated, a TBS-saponin solution was used for all wash steps. The reaction was visualised by using the APAAP (alkaline phosphatase anti-alkaline phosphatase) technique with the bridge antibody, rabbit anti-rat IgG (DAKO, Z0494) and monoclonal APAAP rat IgG (DAKO, D0488), with each antibody being applied for 30 min. To increase the staining intensity, these two steps were repeated. After a final wash with TBS, bound antibodies were visualised using Liquid Permanent Red Chromogen (LPR) (DakoCytomation). Counterstaining was performed as above.

### Quantification

The stained samples were assessed in a blinded fashion using an Axioplan 2 microscope (Carl Zeiss Jena GmbH) at a magnification of x400. To facilitate counting, a graticule was applied to the ocular. Eight representative sections of each of the three lung microenvironments (peribronchial, perivascular and alveolar tissue), were assessed. Positive cells were counted and the data expressed as the number of cells/mm^2^ [11].

### Multiplex cytometric bead assay

Cell lysate of the apical lobe of the mouse lung was used for cytokine measurements. BD Cytometric Bead Array (CBA) Mouse Th1/Th2/Th17 Cytokine kit (BD Biosciences) was used to measure IL-2, IL-4, IL-6, IFN-γ, TNF, IL-17A and IL-10 in the lung tissue. Data were acquired on a FACS ARIA and samples were analysed using FCAP Array Software (BD Biosciences).

### Preparation of tissue for laser capture microdissection (LCM)

The frozen lung tissue is sectioned at 7 µm in a cryostat (Leica Microsystems Nussloch GmbH, Germany) and a minimum of 10 sections per animal was prepared for LCM. The frozen sections were placed on Nuclease Free – MembraneSlides NF 1.0 PEN (Carl Zeiss MicroImaginG GmbH, München, Germany) and then stained with Cresyl Violet acetate (Sigma-Aldrich®) according to the instruction “RNA extraction from frozen section” (PALM Laboratories, ZEISS) . The slides were allowed to air-dry completely before stored desiccated at −80°C to prevent activation of endogenous RNase in the tissues.

### Laser capture microdissection (LCM)

The Carl Zeiss Laser Microdissection and Pressure Catapulting (LMPC) technology (http://PALM-microlaser.com) was used, the specimen was microdissected by a focused laser beam, and then a defined laser pulse transports the cut piece of the specimen out of the object plane into a collection device, AdhesiveCap opaque (Carl Zeiss MicroImaginG GmbH, München, Germany). The lung section to be subjected to LCM was visualised with the PALM- MicroBeam microscope (Carl Zeiss, Germany) at a magnification of x 5. Typically, on each tissue section most of the peribronchial, perivascular and alveolar tissue were obtained and at least 10 sections were used for each mouse. The time required was about 3 h/per section.

### RNA isolation

LCM cells were collected from three different microenvironments of three OVA sensitised and exposed (OVA/OVA) mice. Whole lung tissue section of 50 µm from OVA sensitized and PBS exposed (OVA/PBS) mouse was used as control. The RNA was extracted using a QIAGEN RNeasy® Micro Kit (QIAGEN) according to manufacturer's protocol for the purification of total RNA from microdissected cryosections (QIAGEN). The RNA quality and quantity was determined using an Agilent Bioanalyzer with a RNA 6000 Nano Assay (Agilent Technologies, Deutschland GmbH, Waldbronn).

### Real-time reverse transcription polymerase chain reaction (real-time RT-PCR)

RNA from three OVA/OVA mice were pooled based on three microenvironments whereas RNA from one OVA/PBS mouse was used as a control. Each cDNA reaction was prepared using the SABiosciences's RT^2^ First Strand Kit and 135 ng of total RNA according to the manufacturer's protocol. Real-time RT-PCR was carried out using RT^2^ Profiler™ PCR Array (PAMM-0011D) plates containing 84 mouse inflammatory cytokines, chemokines and receptor genes from Super array Bioscience Corporation, USA. SABiosciences's RT^2^ SYBR Green qPCR Master Mix was used for detection and the array plates were run in a Bio-Rad CFX96 Real-Time PCR Detection System (Bio-Rad) according to manufacturer's instructions (SABioscience). Results were monitored using different controls available on the plates. Gene expression levels were measured by the threshold cycle (Ct) and samples showing more than one peak in the melting curve were excluded from the analysis performed using CFX Manager 2.0 software (Bio-Rad). Twenty-four genes were not considered as they did not pass our quality control, leaving 60 genes for further analysis. Data were normalized with five housekeeping genes [Gusb (Glucuronidase, beta), Hprt1 (Hypoxanthine guanine phosphoribosyl transferase 1), Hsp90ab1 (Heat shock protein 90kDA alpha (cytosolic), class B member 2), Gapdh (Glyceraldehyde-phosphate dehydrogenase), Actb (Actin, beta cytoplasmic)] available in the plates. Finally, fold-changes compared to the control mice were calculated using the manufacturer's software (SABioscience). A fold-change cutoff ≥3 was used to identify molecules whose expression was differentially regulated.

### Bioinformatic Analysis

Genes of interest identified using the RT2 ProfilerTM PCR Array were further analysed by Ingenuity Pathways Analysis (IPA; version 9.0) (Ingenuity® Systems, www.ingenuity.com), specifically in regards to their interactions. The fold change values were compared to the control. IPA utilises the Ingenuity Pathways Analysis Knowledge Base (IPA KB), a manually curated database of protein interactions from the literature, to analyse data. This KB was used to annotate the genes of interest and reveal their associations.

### Data analysis

All data were expressed as mean ± SEM (standard error of the mean). Statistical analyses were carried out using a non-parametric analysis of variance. The Mann-Whitney *U* test was used to determine the significant differences between the individual groups. A *p* value <0.05 was considered statistically significant.

## Results

### Lung eosinophilia after allergen exposure

All animals were sensitised to OVA and exposed to either OVA or PBS on five consecutive days, with the protocol presented in Figure 1A. The number of CD45^+^CCR3^+^SSC^high^ granulocytes, representing mature eosinophils, was significantly increased in the lung tissue after OVA exposure, compared to control PBS exposure (Figure 1B), confirming that a model of allergic airway inflammation had been established.

### Lung CD4^+^ T cell proliferation *de novo* and *in situ* after allergen exposure

**Figure 2:**
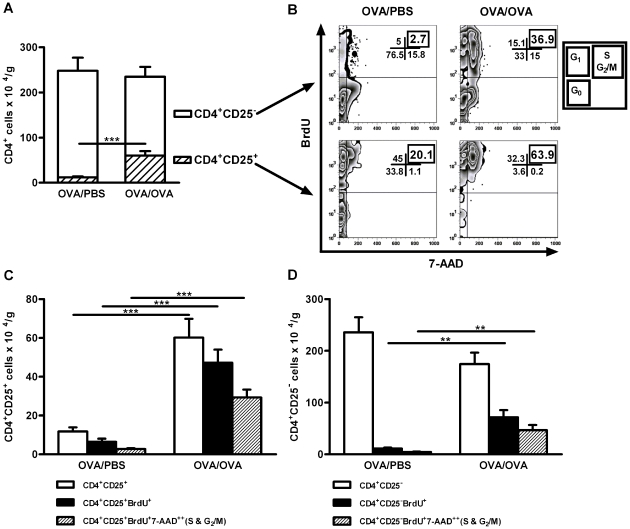
Lung CD4^+^ cells proliferate *de novo* and *in situ* during allergic airway inflammation. A) Total lung CD4^+^ and CD4^+^CD25^+^ cells (per gram tissue) in sensitised mice exposed either to PBS or OVA on five consecutive days. B) Representative FACS plots showing the CD4^+^ CD25**^−^** and CD4^+^CD25^+^ the lung cells that are also positive for BrdU (newly produced) and stained with 7-AAD (DNA) to determine cell-cycle status. Cells (%) are shown for the indicated gates and quadrants. The two upper plots represent CD4^+^CD25^−^ cells, and the two lower plots represent CD4^+^CD25^+^ cells. The left plots represent data from mice exposed to PBS and the right plots represent data from mice exposed to OVA. Within each plot, the G_1_ phase is represented by the upper left quadrant, the G_0_ phase in the lower left quadrant, and the S, G2/M phase in the upper right quadrant. C) Number of CD4^+^CD25^+^ lung cells (per gram tissue) (white column) demonstrating signs of proliferation, as assessed by the incorporation of BrdU (filled column) and BrdU and 7-AAD (hatched columns) after exposure of mice to PBS or OVA. D) Number of CD4^+^CD25^−^ lung cells (per gram tissue) (white column) demonstrating signs of proliferation, as assessed by the incorporation of BrdU (filled column) and BrdU and 7-AAD (hatched columns) after exposure of mice to PBS or OVA. All data are shown as mean ± SEM, n  =  5–8 mice/group (*** *p*<0.001, ** *p*<0.01).

The total number of CD4^+^ T cells in lungs did not significantly differ between OVA and PBS exposed mice (Figure 2A). However, the total number of CD4^+^CD25^+^ cells in the lung significantly increased after repeated OVA exposure (Figure 2A). This finding concurs with other studies, demonstrating that regulatory T cells in the thymus are exclusively CD25^+^, while those in the periphery are both CD25^+^ and CD25**^-^** subsets of mature CD4^+^ T cells [12]–[14]. CD4^+^CD25**^−^** *Foxp3^+^* T cells have indeed been shown to be more effective than CD4^+^CD25^+^Foxp3^+^ T cells in mediating tolerance, emphasising their putative importance *in vivo* [15], [16] and enforcing the importance of determining the presence of both the CD25^+^ and CD25^−^ subsets. The expansion of the CD4^+^CD25^+^ subset of cells could be explained by local proliferation in the lung during repeated OVA exposure. An established FACS method was used to quantify the presence of newly produced cells by measuring the incorporation of BrdU and 7-AAD [17]. Indeed, both CD4^+^CD25**^−^** and CD4^+^CD25^+^ cells proliferated in the lung during allergen exposure (Figure 2C–D), confirming our hypothesis. As the relative number of CD4^+^CD25**^−^** cells is larger in the control group, more CD4^+^CD25**^−^** cells are newly produced (Figure 2D). Thus, cells in both the CD25**^+^** and CD25**^−^** CD4 populations are proliferating in the lung during repeated OVA exposure (S and G_2_/M phase) (Figure 2B). Importantly, comparison of the proliferation rate *de novo* and *in situ* revealed a greater number of proliferating CD4^+^CD25^−^ T cells (64%) vs. CD4^+^CD25^+^ T cells (37%) in the lung during allergen exposure (Figure 2B).

### The expression of T cell transcription factors, T-bet^+^(Th1), GATA-3^+^( Th2), RORγt^+^(Th17) and Foxp3^+^(Treg) cells in CD4^+^CD25^+^ cells in the lung tissue increased after allergen exposure

**Figure 3:**
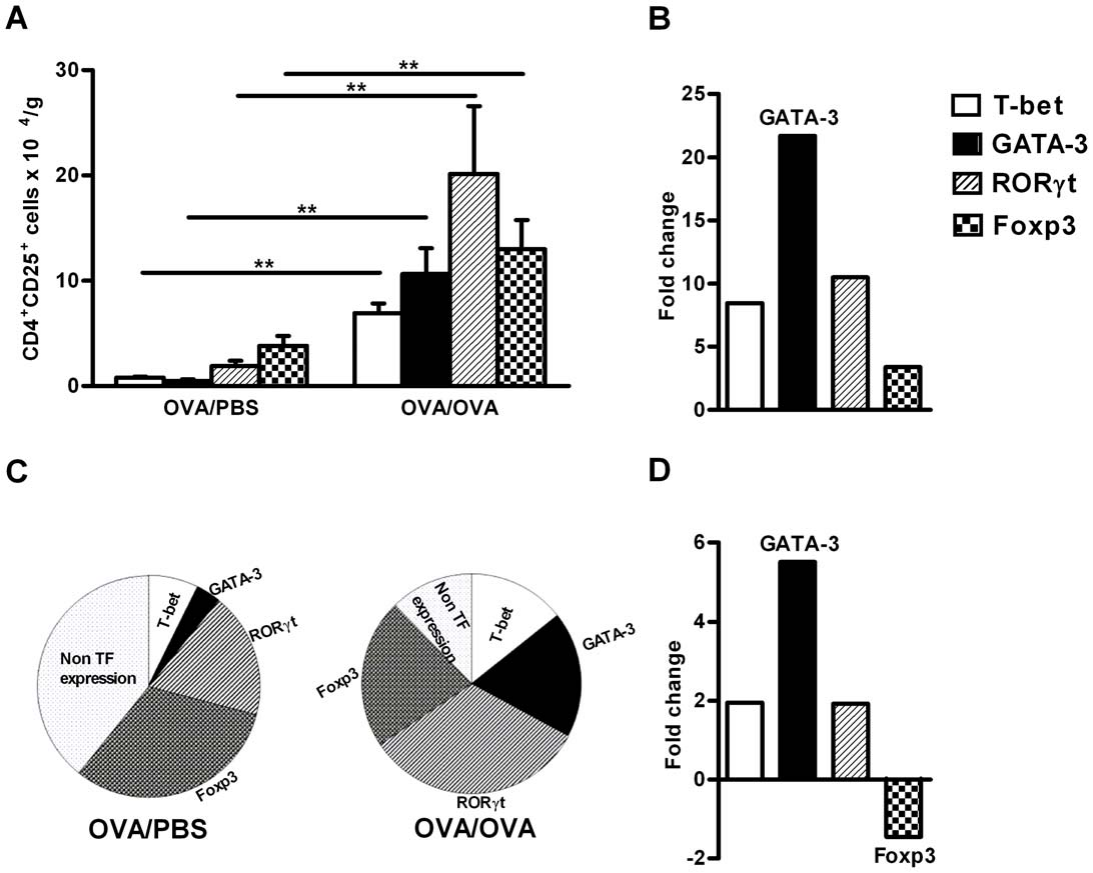
Expression of T cell transcription factors, T-bet^+^(Th1), GATA3^+^(Th2), RORγt^+^ (Th17) and Foxp3^+^(Tregs) cells in the CD4^+^CD25^+^ cells in lung during allergic inflammation. A) Number of lung CD4^+^CD25^+^ cells (per gram tissue) expressing T-bet, GATA-3, RORγt and Foxp3 after exposure of mice to PBS or OVA. Data is shown as mean ± SEM, n =  5–8 mice/group (*** *p*<0.001, ** *p*<0.01). B) Fold change of the total number of lung cells expressing the four different transcription factors (T-bet, GATA-3, RORγ and Foxp3) in CD4^+^CD25^+^ cells after exposure of mice to OVA compared to PBS. C) Pie chart showing the relative presence of Th1 (T-bet^+^) ,Th2 (GATA-3^+^),Th17 (RORγt^+^ ) and Treg (Foxp3^+^) T cells expressed as percentage of CD4^+^CD25^+^ cells in OVA and PBS exposed animals. TF = transcription Factor. D) The fold change of the relative presence of Th1 (T-bet^+^) ,Th2 (GATA-3^+^),Th17 (RORγt^+^ ) and Treg (Foxp3^+^) T cells expressed as percentage of CD4^+^CD25^+^ cells after exposure of mice to OVA compared to PBS

The presence of different effector T cells and Treg cells within the CD4^+^CD25^+^ cell compartment was determined by FACS evaluation of the expression of specific transcription factors. The number of Th1 (CD4^+^CD25^+^T-bet^+^), Th2 (CD4^+^CD25^+^GATA-3^+^), Th17 (CD4^+^CD25^+^RORγt^+^) and Treg (CD4^+^CD25^+^Foxp3^+^) cells were all significantly expanded in the lung tissue during allergen exposure (Figure 3A). Interestingly, the majority of the expanded cells are Th17 cells, followed by Treg, Th2 and Th1 cells. However, when determining the fold increase of Th1, Th2, Th17 and Treg cell numbers (OVA-OVA/OVA-PBS) we discovered that the GATA-3 expressing Th2 cells were increased up to 22 times more compared to any of the other effector T cells (Figure 3B).

As all of the T effector cells are generated from common naïve cells we were interested to evaluate the relevance between them. The relative change of cells, expressed as a percentage of all CD4^+^ cells, revealed substantial differences between PBS and OVA exposed animals (Figure 3C). For example, a considerable percentage of CD4^+^ cells in OVA/PBS exposed animals did not express any of above transcription factors, which was lower in OVA exposed animals. Among the studied transcription factors, GATA-3, changes drastically in response to OVA exposure, with a 6 fold increase compared to PBS exposure, whereas Foxp3 was decreased (Figure 3D).

### The expression of T cell transcription factors, T-bet^+^(Th1), GATA-3^+^(Th2), RORγt^+^(Th17) and Foxp3^+^(Treg) cells in CD4^+^CD25^−^ cells in the lung tissue increased after allergen exposure

**Figure 4:**
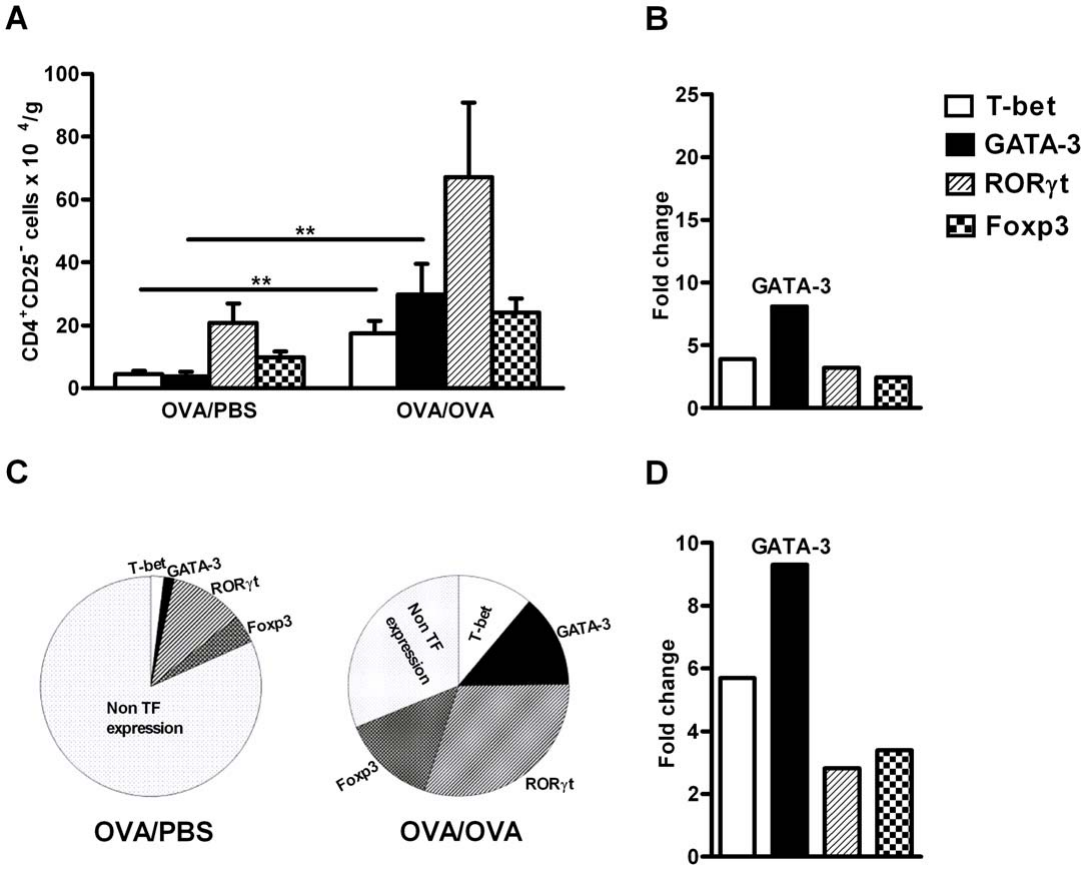
Expression of T cell transcription factors, T-bet^+^(Th1), GATA3^+^(Th2), RORγt^+^(Th17) and Foxp3^+^(Tregs) cells in the CD4^+^CD25^−^ cells in lung during allergic inflammation. A) Number of lung CD4^+^CD25^−^ cells (per gram tissue) expressing T-bet, GATA-3, RORγt and Foxp3 after exposure of mice to PBS or OVA. Data is shown as mean ± SEM, n =  5–8 mice/group (*** *p*<0.001, ** *p*<0.01). B) Fold change of the total number of lung cells expressing the four different transcription factors (T-bet, GATA-3, RORγ and Foxp3) in CD4^+^CD25^−^ after exposure of mice to OVA compared to PBS. C) Pie chart showing the relative presence of Th1 (T-bet^+^), Th2 (GATA-3^+^), Th17 (RORγt^+^) and Treg (Foxp3^+^) T cells expressed as percentage of CD4^+^CD25^−^ cells in OVA and PBS exposed animals. TF = transcription Factor. D) The fold change of the relative presence of Th1 (T-bet^+^), Th2 (GATA-3^+^), Th17 (RORγt^+^ ) and Treg (Foxp3^+^) T cells expressed as percentage of CD4^+^CD25^−^ cells after exposure of mice to OVA compared to PBS.

Expression of transcription factors for Th1, Th2, Th17 and Treg cells was observed also in CD4^+^ cells lacking the IL-2Rα (the CD4^+^CD25**^−^** population; Figure 4). The number of Th1 and Th2 cells in the CD4^+^CD25^−^ cells was significantly increased in the lung tissue after allergen exposure, while the number of Th17 and Treg cells also numerically increased, but not significantly. In the CD4^+^CD25**^−^** cell population, the majority of the cells express transcription factors for Th17 cells, followed by Th2, Tregs and Th1 cells (Figure 4A). Evaluation of the fold increase of the cell numbers revealed that in CD4^+^CD25**^−^** cells, the Th2 cells dominate, however to a smaller magnitude vs CD4^+^CD25^+^ (Figure 4B). When the relevant expression of all transcription factors in CD4^+^ cells was calculated, relatively fewer cells express the transcription factors for Th1, Th2, Th17 and Treg cells in OVA/PBS animal compared to OVA/OVA animals (Figure 4C). Calculation of the fold increase also showed that even in the CD4^+^CD25**^−^** population, GATA-3 was more increased compared to the changes observed for T-bet, RORγt and Foxp3( Figure 4D).

### Lung Th1, Th2, Th17 and Treg cells proliferate during allergic airway inflammation in both the CD4^+^CD25^+^ and CD4^+^CD25^−^ cell populations

**Figure 5:**
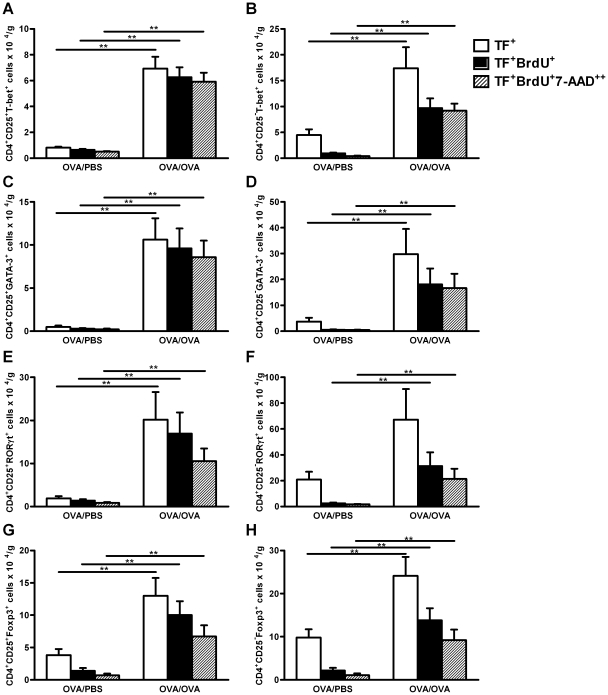
Lung Th1, Th2, Th17 and Treg cells in CD4^+^CD25^+^ and CD4^+^CD25^−^ populations proliferate *de novo* and *in situ* during allergic inflammation. Number of lung CD4^+^CD25^+^ (A, C, E and G) and CD4^+^CD25^−^ cells (B, D, F, H) that are expressing transcription factors (TF); T-bet (A and B; Th1), GATA-3 (C and D; Th2), RORγt (E and F; Th17) and Foxp3 (G and H; Treg) and have incorporated BrdU (filled column) or have incorporated BrdU and also express a double amount of DNA (7-AAD^++^; hatched columns) in mice exposed to PBS or OVA. Data are shown as mean number of cells per gram tissue +SEM. ** *p*<0.01.

As all effector T cells and Treg cells investigated increased during allergic inflammation, the degree of ongoing proliferation was also assessed in each cell type, by determining the percentage of newly produced cells and their cell cycle phase. Allergen exposure significantly increased the production of new Th1, Th2, Th17 and Treg cells in both CD25^+^ and CD25**^−^** populations (Figure 5A–H). However, when the percentage of newly produced cells in each population is compared, more than 90% of the Th1, Th2 and Th17 cells (Figure 5A,C,E) and about 80% of the Treg cells (Figure 5G) are newly produced in the CD4^+^CD25^+^ population, while only about 60% of the Th1, Th2 and Treg cells(Figure 5B,D,H) and 50% of Th17 cells (Figure 5F) are newly produced in CD4^+^CD25**^−^** population, suggesting that the presence of the IL-2Rα increases the proliferative activity of these cells. When the number of newly produced cells in the S-G2/M phase is examined, more than 95% of the newly produced Th1 and Th2 cells in both CD25^+^ and CD25**^−^** populations were proliferating *in situ*, compared to only 65% of the newly produced Th17 and Treg cells in CD25^+^ and CD25**^−^** populations. Together, these data show that the majority of Th1, Th2, Th17 and Treg cells in CD25^+^ population are newly produced after allergen exposure, whereas only half of the Th1, Th2, Th17 and Treg cells in CD25**^−^** population are newly produced after exposure. This confirms the proliferative enhancement in the presence of IL-2 receptor in the lung during allergen exposure.

Next we estimated the Th1, Th2, Th17 and Treg cells presence in the CD4^+^ cell pool. T effector cells in S and G_2_/M phase i.e.: Th1 (CD4^+^CD25^+/−^Tbet^+^BrdU^+^7-AAD^++^), Th2 (CD4^+^CD25^+/−^ GATA-3^+^BrdU^+^7-AAD^++^), Th17 (CD4^+^CD25^+/−^RORγt^+^BrdU^+^7-AAD^++^) and Treg (CD4^+^CD25^+/−^Foxp3^+^BrdU^+^7-AAD^++^) cells are expressed as % of total CD4^+^ T cells. Among T effector cells, the Th2 cells had the higher fold increase after allergen exposure in both CD25^+^ (Th1: 11, Th2: 31, Th17: 8, and Treg: 10) and CD25^−^ (Th1: 10, Th2: 23, Th17: 9, and Treg: 10 respectively).

### The distribution of T cell transcription factors differs in the lung tissue during allergic airway inflammation

**Figure 6:**
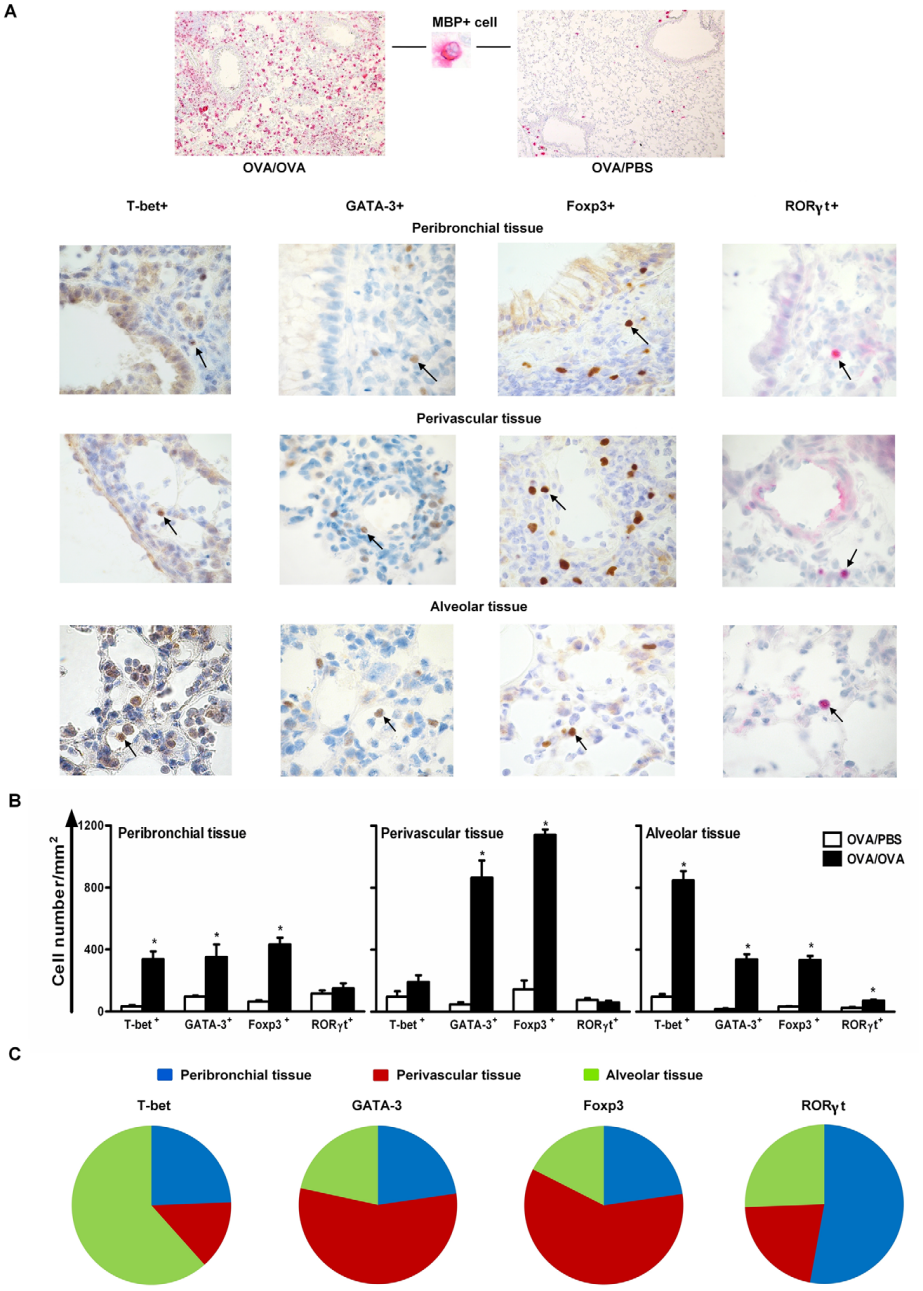
The distribution of T cell transcription factors differs in the lung microenvironments during allergic inflammation. A) Photographs of immunohistochemistry of MBP (red) in OVA or PBS exposed sensitised mice, T-bet, GATA-3, Foxp3 (brown) and RORγt (red) in peribronchial, perivascular and alveolar tissue after exposure to OVA in sensitised mice. B) Quantification (cells/mm^2^) of T-bet, GATA-3, Foxp3 and RORγt expressing cells in different compartments of the lungs, including peribronchial tissue, perivascular tissue and alveolar tissue, after exposure to PBS or OVA. Data shown as mean+SEM, *<0.05. C) Pie chart showing the relative distribution of T-bet, GATA-3, Foxp3 and RORγt expressing cells in the different lung compartments after exposure of mice to OVA.

As shown by FACS, all of the investigated T effector cells and Treg cells in the lung were increased in numbers, with many in a proliferative state, during allergic inflammation. It has been reported that T cells can traffic into the inflamed lung tissue during allergic inflammation, but their micro environmental distribution has so far not been investigated. Therefore, we stained lung tissue from OVA/PBS and OVA/OVA mice for T-bet (Th1), GATA-3 (Th2), Foxp3 (Treg cells) and RORγt (Th17) expressing cells by immunohistochemistry (Figure 6A), and quantified their respective presence in three different areas of the lung, including lung peribronchial tissue, lung perivascular tissue and lung alveolar tissue. The immunohistochemical staining of different transcription factor positive cells is documented in the nucleus in the three different lung compartments of OVA /OVA animals (Figure 6A). In addition, also MBP^+^ cells, representing eosinophils, were present in the lung tissue of allergen exposed mice (Figure 6A). The mice acquired a significant accumulation of T-bet^+^, GATA-3^+^ and Foxp3^+^ cells in the peribronchial, perivascular and alveolar lung tissue after allergen exposure, while RORγt^+^ cells increased only in the alveolar tissue, but not in the peribronchial and perivascular tissue (Figure 6B). Focusing in T effector cell relevant distribution, T-bet^+^ cells were predominantly distributed in the alveolar lung tissue, followed by the peribronchial and perivascular tissue. GATA-3^+^ and Foxp3^+^ cells were mainly expressed in the perivascular tissue, followed by peribronchial and alveolar tissue. Although the RORγt^+^ cells were the smallest population detected in lung tissue, most were distributed in the peribronchial tissue, followed by the alveolar and perivascular tissue (Figure 6C).

### Cytokine concentration in lungs during allergic inflammation

**Figure 7:**
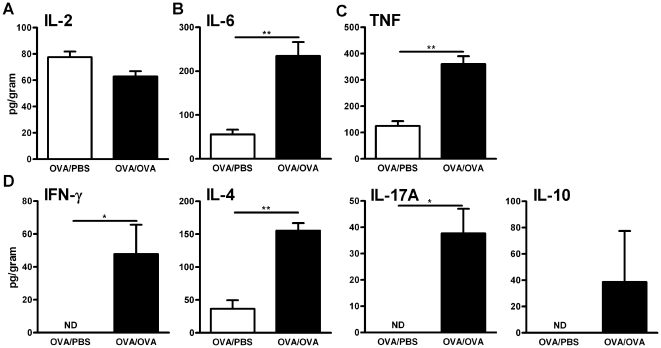
Cytokine concentration in lung during allergic inflammation. Concentration of IL-2(A), IL-6(B), TNF (C), IFN-γ, IL-4, IL-17A and IL-10 (D) was assessed using CBA; Results are expressed as pg/gram lung tissue, in mice exposed to PBS or OVA. Data are shown as mean ± SEM, *p<0.05, **p<0.01.

To determine the general inflammatory milieu in the lung, pro and anti-inflammatory cytokines, as well as cytokines relevant to the different T helper cell subsets, were quantified in the lung supernatant. Specifically, IL-2, IL-6, TNF (tumour necrosis factor), IFN-γ (Th1), IL-4 (Th2), IL-17A (Th17) and IL-10 (Treg) were quantified after PBS or OVA exposure. No differences were found in the expression of IL-2 (Figure 7A), whereas both IL-6 and TNF were significantly increased after allergen exposure, indicating a general inflammatory status (Figure 7B-C). IFN-γ, IL-4 and IL-17A were all significantly increased, with IL-4 the most prominently increased, indicating a Th2 activity in the tissue (Figure 7D). This supports the presence of a Th2-polarised inflammatory process in the lung environment. It should be noted that a number of cells, both structural as well as leukocytes have the capacity to release many of these cytokines.

### Evaluation of the gene expression in peribronchial, perivascular and alveolar lung tissue as a marker of local inflammatory response

**Figure 8:**
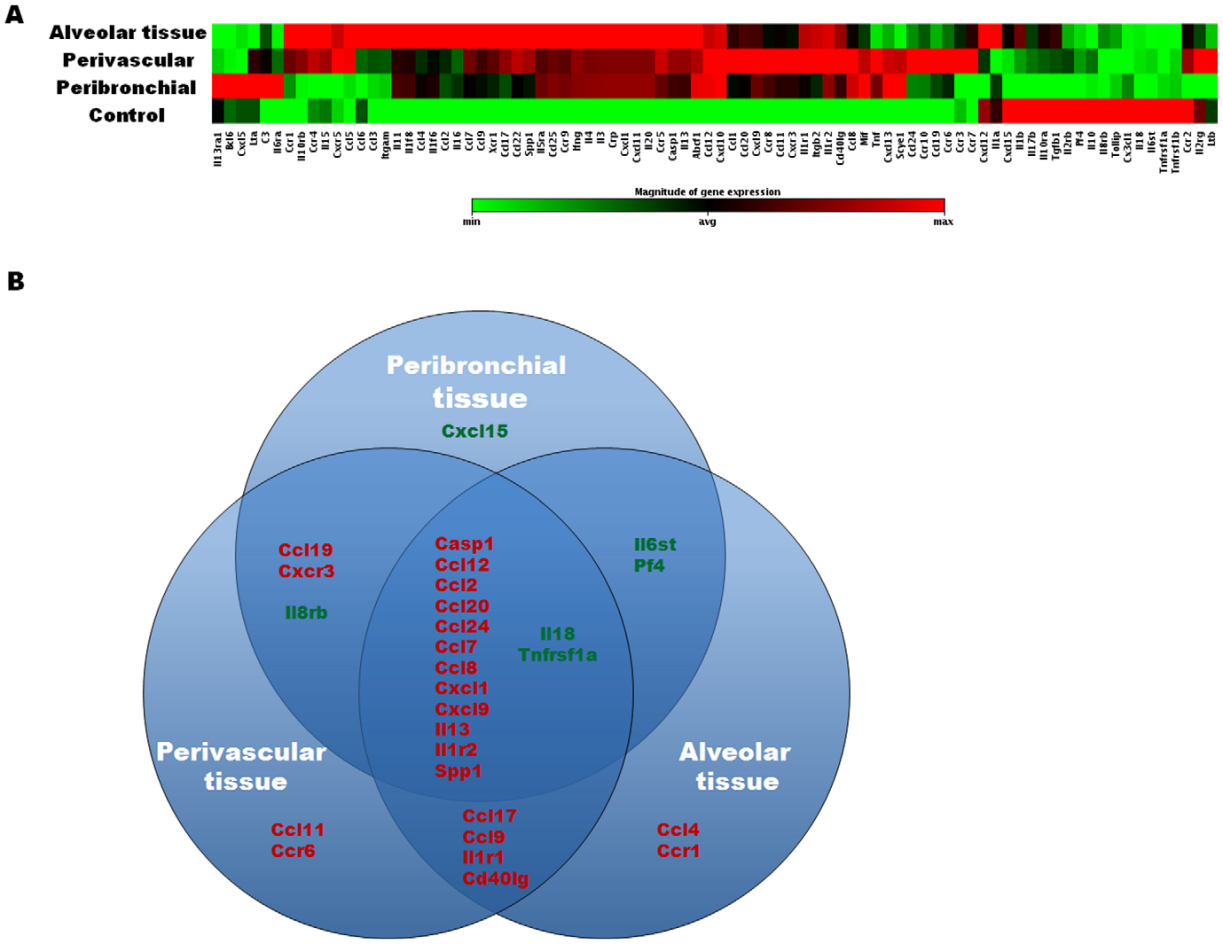
Expression of inflammatory cytokine, chemokine and receptor genes in the lung after allergen exposure compared to control. A) The different lung tissue compartments (alveolar, perivascular and peribronchial) were isolated from lung tissue from OVA or PBS exposed mice using the PALM microBeam microscopy. Lung compartment samples were taken from three different animals and then pooled. RNA was extracted and the inflammatory response in each microenvironment was assessed using real-time RT-PCR array of 84 inflammatory cytokines, chemokines and receptors. The expression of these genes in each compartment is visualized as heat map. Red colour indicates up-regulated genes, green colour indicates down-regulated genes, and the dark colour indicates great fold change. B) Venn diagram of genes with fold change ≥3 times after allergen exposure in at least one tissue compartment compared to control. Up-regulated genes are shown in red font and down regulated genes are shown in green font.

To further evaluate the local inflammatory microenvironment in the different lung tissue compartments, we used laser capture microdissected peribronchial, perivascular and alveolar tissue from OVA/OVA mice and control mice, followed by a real-time RT-PCR analysis and quantification of expression of 60 different cytokines, chemokines or respective receptor genes (Figure 8A). The effect of allergen exposure in all three tissue compartments was expressed as fold change compared to gene expression in OVA/PBS animals (OVA-OVA/OVA-PBS). Allergic inflammation resulted in a wide variation of inflammatory response from down-regulation (*i.e.* Il8>8 times in peribronchial tissue) to up-regulation (*i.e.* more than 20 fold for Ccl8 in perivascular tissue) (Table 1).

**Table 1:** Fold change of gene expression in peribronchial, perivascular and alveolar lung tissue of OVA exposed mice compared to PBS control.

RefSeq*	Symbol	Peribronchial	Perivascular	Alveolar tissue
NM_013854	Abcf1	2,17	2,03	2,22
NM_009744	Bcl6	1,21	−1,09	−1,09
NM_009778	C3	1,65	1,34	1,22
NM_009807	Casp1	4,96	6,05	7,36
NM_011329	Ccl1	1,60	2,25	1,73
NM_011330	Ccl11	2,95	4,34	2,73
NM_011331	Ccl12	3,86	3,94	3,63
NM_011332	Ccl17	2,08	3,41	4,08
NM_011888	Ccl19	3,03	6,67	1,84
NM_011333	Ccl2	4,86	5,73	12,13
NM_016960	Ccl20	5,13	9,11	6,23
NM_019577	Ccl24	3,92	13,07	3,63
NM_013652	Ccl4	2,87	2,48	4,56
NM_013653	Ccl5	1,05	1,96	1,97
NM_009139	Ccl6	−1,47	−1,04	1,64
NM_013654	Ccl7	8,57	11,62	15,78
NM_021443	Ccl8	18,38	20,08	11,08
NM_011338	Ccl9	2,53	3,03	4,23
NM_009912	Ccr1	1,51	2,69	3,39
NM_009915	Ccr2	−2,07	−1,11	−1,32
NM_009914	Ccr3	−1,01	1,10	1,04
NM_009916	Ccr4	−1,17	1,42	1,51
NM_009835	Ccr6	2,22	3,83	1,79
NM_009913	Ccr9	2,38	2,29	2,89
NM_009142	Cx3cl1	−1,66	−2,06	−2,03
NM_008176	Cxcl1	5,13	4,95	6,23
NM_021704	Cxcl12	−1,57	−1,22	1,09
NM_011339	Cxcl15	−3,14	−2,81	−1,48
NM_019932	Pf4	−4,29	−2,62	−3,53
NM_009141	Cxcl5	2,08	−2,32	−1,95
NM_008599	Cxcl9	5,10	6,81	4,69
NM_009910	Cxcr3	2,99	4,43	2,62
NM_008348	Il10ra	−1,65	−1,37	−1,21
NM_008349	Il10rb	1,01	1,35	1,46
NM_008355	Il13	4,08	4,95	6,02
NM_133990	Il13ra1	1,44	−1,55	−1,77
NM_008357	Il15	−1,18	1,32	1,42
NM_008360	Il18	−8,11	−5,99	−6,68
NM_010554	Il1a	−1,06	−1,19	1,11
NM_008361	Il1b	−2,50	−1,95	−1,21
NM_008362	Il1r1	2,36	3,63	3,27
NM_010555	Il1r2	3,05	3,63	3,46
NM_008368	Il2rb	−2,46	−1,59	−2,03
NM_013563	Il2rg	−1,62	1,16	−1,23
NM_008370	Il5ra	2,14	2,07	2,60
NM_010560	Il6st	−3,18	−2,74	−3,20
NM_009909	Il8rb	−6,15	−6,37	−2,87
NM_008401	Itgam	1,07	1,25	1,75
NM_008404	Itgb2	1,80	2,88	2,69
NM_010735	Lta	1,33	1,12	−1,21
NM_008518	Ltb	−1,26	1,33	−1,17
NM_010798	Mif	1,92	1,80	1,36
NM_007926	Scye1	1,56	1,49	1,01
NM_009263	Spp1	5,86	9,63	11,55
NM_011577	Tgfb1	−2,38	−1,62	−1,30
NM_011609	Tnfrsf1a	−3,07	−3,64	−3,61
NM_011610	Tnfrsf1b	−2,28	−2,33	−2,64
NM_011616	Cd40lg	2,79	3,97	3,39
NM_023764	Tollip	−1,68	−1,81	−1,58
NM_011798	Xcr1	1,20	1,31	1,45

Genes showed have fulfilled all quality criteria, including Ct value, melting curve and melting temperature.

*RefSeq, reference sequence.

Only genes that were changed ≥3 times in at least one tissue compartment were considered having biological importance and were involved in a further analysis. Firstly, we evaluated genes affected by allergic inflammation (Figure 8B). We found that 22 genes were upregulated and 6 genes were downregulated after allergen exposure. Fourteen of these were changed in all three compartments. Of these, twelve genes were upregulated (Casp1, ccl12, ccl20.Ccl24, Ccl7, Ccl8, Cxcl1, Cxcl9, Il13, Il1r2 and Spp1) and two were downregulated (Il18 and Tnfrsf1a). Peribronchial and perivascular tissue had change in expression of three genes in common (Ccl19 and Cxcr3 were upregulated Il-8rb was downregulated), while peribronchial and alveolar tissue had changed expression in two gene (Il-6st and Pf4/Cxcl4), both downregulated. Alveolar and perivascular microenvironments shared change in expression of four genes (Ccl17, Ccl9, Il1r1 and Cd40lg) that were all upregulated. Finally, there were genes that changed only in one compartment, which included upregulation Ccl11 and Ccr6 in the perivascular compartment and Ccl4 and Ccr1 in the alveolar compartment, and downregulation of Ccxl15 in the peribronchial compartment (Figure 8B).

Next we focus on the differences between the three compartments. For analysis we grouped genes in three categories; genes involved in general inflammation, genes involved in allergic inflammation and genes from chemokines that can affect T effect cells migration, as reported in the literature, followed by analysis of their relationship using Ingenuity Pathways Analysis.

**Figure 9:**
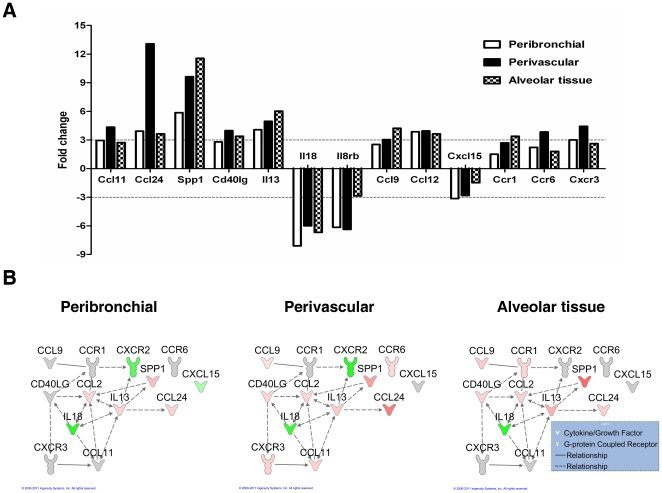
Gene expression in lung microenvironments during allergic inflammation. A) Fold change of genes related to allergic inflammation in peribronchial, perivascular and alveolar tissue. Fold change is calculated by comparing OVA exposed mice to PBS control. 3 fold change threshold indicated by dotted line was considered biological relevant. B) Network of differentially expressed genes in OVA exposed mice compared to the PBS exposed controls. A network of genes associated with allergic inflammation, generated by Ingenuity Pathways Analysis (3.0 fold change threshold), of OVA/OVA mice compared to OVA/PBS mice. The expression of these genes in three lung microenvironments was examined; peribronchial, perivascular and alveolar tissue. Lines indicate relationships between molecules (solid line = direct interaction; dashed line = indirect interaction). Arrows at the end of these lines indicate the direction of the interaction. Molecules that are up-regulated and down-regulated in the dataset are coloured red and green respectively. Darker colouring of the molecules indicates a higher fold change. Grey molecules did not meet the threshold of 3 fold change. Uncoloured molecules have been added from the Ingenuity Knowledge Base. The exact fold change values of each molecule are shown in Table 1.

To determine the role in allergic inflammation, 13 genes related to allergy were included (Figure 9A), revealing differences between the three compartments. Among them, eotaxin-2 (Ccl24) was the highest amplified gene and was expressed mainly in perivascular tissue. Eotaxin-1(Ccl11) was amplified in the same manner, but to a smaller degree. The main Th2 cytokine, Il13, was also expressed in all three compartments with emphasis in alveolar tissue. This was also seen for Spp1 (osteopontin), which showed a greater increase compare to Il13. Il18 was downregulated in all compartments, while Il8rb (Cxcr2) was downregulated mainly in the peribronchial and perivascular areas. Direct and indirect relationships between some of these allergy-associated genes was documented (Figure 9B).

**Figure 10:**
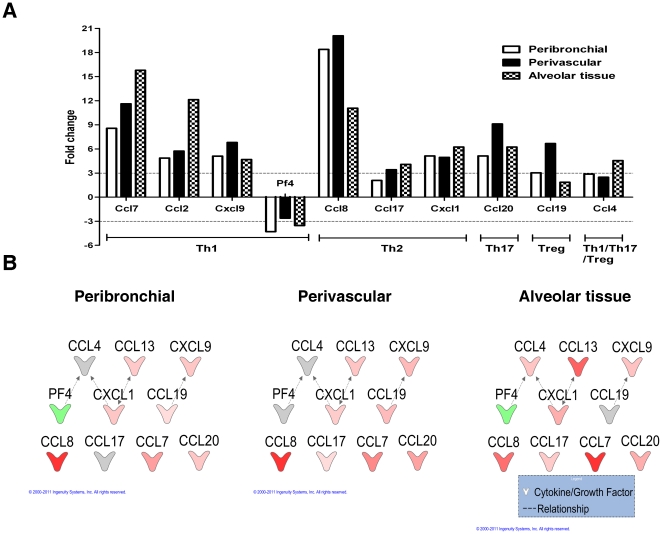
Chemokines related to Th1, Th2, Th17 and Treg cells during allergic inflammation. A) Fold change of genes related to chemoattractants to Th1, Th2, Th17 and Treg cells in peribronchial, perivascular and alveolar tissue. Fold change is calculated by comparing OVA exposed mice to PBS control. 3 fold change threshold indicated by dotted line was considered biological relevant. B) Network of differentially expressed genes in OVA exposed mice compared to PBS exposed controls. A network of chemokines, generated by Ingenuity Pathways Analysis (3.0 fold change threshold), of OVA/OVA mice compared to OVA/PBS mice. The expression of these genes in three lung microenvironments was examined; peribronchial, perivascular and alveolar tissue. Lines indicate relationships between molecules (dashed line = indirect interaction). Arrows at the end of these lines indicate the direction of the interaction. Molecules that are up-regulated and down-regulated in the dataset are coloured red and green respectively. Darker colouring of the molecules indicates a higher fold change. Grey molecules did not meet the threshold of 3 fold change. Uncoloured molecules have been added from the Ingenuity Knowledge Base. The exact fold change values of each molecule are shown in Table 1.

Our final goal was to describe the expression of chemokine genes that may have a role in the homing of Th1, Th2, Th17 and Treg cells to different compartments. The Th1 related chemokines Ccl7, Ccl2 and Cxcl9 were increased in all three compartments after allergen exposure, with two of them increased in alveolar tissue (Ccl7, Ccl2). Interestingly Pf4 (Cxcl4) was down-regulated. Among the Th2 related chemokines, Ccl8 showed the higher fold increase mainly in perivascular and then in peribronchial tissue. For Th17, Ccl20 was increased mainly in perivascular tissue. The Ccl4 a chemokine (Il17-related) was increased mainly in alveolar tissue. Finally Ccl19, a Treg cell related chemokine was increased mainly in perivascular tissue supporting immunohistochemistry data (Figure 10A).

Analysis with ingenuity showed also a complex relation between the majority of above chemokines, supporting the concept of a sophisticated network of high complexity and high complexity and interaction (Figure 10B).

## Discussion

Our current study shows that airway exposure to allergen in sensitised mice expands all studied effector T cells in the lungs, including T-bet^+^, GATA-3^+^, RORγt^+^ and Foxp3^+^ cells , both in those cell populations expressing the IL-2Rα (i.e. CD25) and those that do not. Among these, the GATA-3 effector Th2 cell is the one most prominently increased after allergen exposure, followed to a lesser extent by the T-bet^+^ Th1 and the RORγt^+^ Th17 cells. In contrast, the Foxp3^+^ Treg cells increased in absolute cells numbers in the CD4^+^CD25^+^ and CD4^+^CD25^-^ population but decreased compare to other T effector cells in the CD4^+^CD25^+^ fraction. All effector T cells are produced *de novo* and proliferate *in situ* in the lungs, but with different profiles. Th1 and Th2 cells proliferate more extensively than Th17 and Treg cells during allergic inflammation. Allergen exposure subsequently results in an accumulation of Th1, Th2 and Treg cells in the peribronchial tissue, as well as an increase in the alveolar lung tissue of Th17 cells. In perivascular tissue, only Th2 and Treg cells increase. A general inflammatory milieu in the lung was showed to involve Th2 cytokines such as IL-4 as well as Th1 cytokines including IFN-γ, and the Th17 cytokine IL-17 as well as several pro-inflammatory cytokines including IL-6. The low concentration of IL-10 argues for a relatively low activity of Treg cells. Finally, allergen exposure induced a wide range of inflammatory genes in lung, whereas some of them only in specific tissues, arguing for the importance of the local microenvironment. Several chemokine genes supported the accumulation pattern of T effector cells.

As far as we know this is the first time that all mentioned T cells are quantified simultaneously in allergic airway inflammation, especially as both their numbers and their topographical localizations are determined, as well as their proliferative status. Furthermore, this is also the first time that the local inflammatory milieu in three main lung tissue compartments, i.e. peribronchial, perivascular and alveolar tissue, is described in relation to the relative expression of a substantial number of relevant pro- and anti-inflammatory genes.

CD25 (IL-2Rα) expression is induced upon T cell activation to form part of the trimeric, high affinity IL-2R complex in combination with CD122 (IL-2Rβ) and CD132 (IL-2Rγ) and has commonly been used as a marker for activated T cells [18]. CD25 is involved in T cell proliferation, activation induced cell death, as well as the actions of both Treg and effector T cells [19], [20]. Soper *et al*. have shown that transgenic mice lacking IL-2Ra have unchanged amounts of Tregs in periphery compared to wild type mice, while mice lacking IL-2Rβ did not, suggesting that IL-2 can be effective even without expression of CD25[21]. These findings were confirmed in a recent human study, where the administration of a humanized anti-CD25 antibody in children undergoing liver transplantation did not affect the circulating Tregs, proposing that the needed IL-2 signal could be mediated by the IL-2 βγ dimeric receptor [22]. Together, these findings argue that the CD25^−^ cells may still use the IL-2 cytokine for their maintenance. However, it is not known whether they are as effective as the CD25^+^ cells in regulating immune response. Furthermore, CD25^−^ cells may have other important functions. For example, CD4^+^CD25^−^Foxp3^+^ cells has been suggested to be more effective than CD4^+^CD25^+^Foxp3^+^ cells in delivering T-cell mediated tolerance [15]. Similarly, CD4^+^CD25^−^ peripheral Treg cells showed preventive functions in the induction of autoimmune responses [16]. These previous findings urged us to quantify not only the CD25^+^ CD4 cells, but also the CD25^−^ fraction of CD4^+^ T cells. In this study, allergen exposure increase the number of CD4^+^ cells that express CD25 from 5% to approximately 25% of CD4^+^ cells in the lungs, confirming its role in allergic inflammation. Importantly, both the CD25^+^ and the CD25^−^ populations are proliferating in the lung, although more prominently in the CD25^+^ cell population, arguing for a key role of IL-2 in this specific cellular process. An explanation of the proliferation of the CD25^−^ cells could be that the cells have shed their IL-2 receptor after having unchanged proliferation. Indeed, CD25 is up-regulated on recently activated effector T cells, but this molecule is subsequently released into the microenvironment [23].

During the past two decades, the Th1/Th2 paradigm of a balance to regulate development of allergy has prevailed, and these two cell types have been implicated in the pathogenesis of different inflammatory diseases including asthma. However, recent evidence suggests that the dichotomous view of Th-cells differentiation must be broadened to several other T-cells. In fact, IL-17-producing Th17 cells appear to play a critical role in autoimmunity and neutrophilic inflammation [24]. Furthermore, anti-inflammatory processes have been suggested to involve Treg cells that suppress the functional activity of other effector T-cells [25]–[27]. However, their relative presence in lung tissue in allergic inflammation has not previously been described. The current study is thus the first to document the changes in Th1, Th2, and Th17 and Treg cells in the lung during allergic airway inflammation. Interestingly, all cells increase in number, but most prominently the Th2 cells, support their major functional role in allergic inflammation.

Even though a relative reduction of Foxp3 cells in the CD4^+^CD25^+^ fraction was observed after allergen exposure, it should be noted that the total number of Foxp3 cells indeed are increased. Importantly, in the CD4^+^CD25^−^ fraction, the Foxp3^+^ cells are increased both as total and relative numbers. It has been argued that attenuation of Foxp3 can cause Treg to revert back to effector T cells, for example Th2-like cells, arguing for plasticity in this population [28], [29]. In the case of Th1 and Th17 cell populations, both of these change in similar patterns during allergen exposure. One interesting finding is that the relative expression of the studied transcription factors is quite small in the CD4^+^CD25^−^ population, as a majority of cells are negative for any of the transcription factors T-bet, GATA3, RORγt and Foxp3 in control animals. However, despite this low baseline expression, the expression of these transcription factors is relatively increased during allergen exposure. Additional T helper subsets are being suggested, and were not identified in the current study, including Th9 [30], Tr1 [31], Th3 [32] and Th22 cells [33], and some of these may indeed contribute to the fraction of CD4^+^CD25^+^ and CD4^+^CD25^−^ that do not express any of the currently studied transcription factors. Clearly, also some of these cells may be involved in the inflammatory process in this model of allergic airway inflammation, and warrant investigation in future studies.

All of the effector T cell- and Treg cell- subsets increase their proliferation in lung during allergen exposure (Figure 5), showing by uptake of BrdU. However, the CD4 cells that were CD25^+^ proliferated to a greater extent than the CD25^−^ cells, indicating a difference in their proliferative ability. Thus, the lung itself is a microenvironment in which all the different studied T-lymphocytes can proliferate. This is documented not only through BrdU incorporation, but also by cell cycle analysing with 7-AAD, measuring total amount of DNA in cells (Figure 5), indicating that many inflammatory cells are in S-phase or G2/M-phase in the lung during allergic inflammation. Even more, the Th2 cells showed the greatest increase in proliferation after allergen exposure, arguing again that these cells are the dominating effector T cells in allergic inflammation.

Several mechanisms for suppression of T effector cells have been proposed. Recent observations have suggested that there could be interaction between different T subsets or with other inflammatory cells, with or without direct cell-to-cell contact [34]–[37]. T cells play a role in both endothelium and epithelium. Our data showing that allergen exposure result in an increased accumulation of T-bet^+^, GATA-3^+^ and Foxp3^+^ cells in peribronchial tissue, argue that these cells may interact in this microenvironment. By contrast, T-bet^+^ cells accumulate to a significant extent also in alveolar lung tissue, and to a lesser extent in perivascular tissue. Further, GATA-3^+^ and Foxp3^+^ cells are accumulated primarily in perivascular tissue. These differences in distribution of T cells argue for the different homing process of these cells to the different microenvironment, which is a unique contribution of the current study to the literature.

The lung tissue represents a highly complicated and sophisticated network of many different cells that can react differently to a variety of stimuli, resulting in unique local inflammatory milieus of cytokines, chemokines and mediators in different microenvironments. The inflammation itself can of course affect different subsets of Th-cells that accumulate in different tissue. For example, it has been shown that NO production from human airway epithelium can affect the Th1/Th2 balance [38]. Another possible mechanism can be that the different T-helper cells that have accumulated in the lungs further drive the inflammation by further release of cytokines. Lordan *et al*. have shown that addition of Th2 cytokines in an already established allergic environment have the potential to sustain further airway inflammation [39].

To investigate the role of cytokines present in the airways, cytokines were first quantified in whole lung, showing that allergen exposure significantly increased levels of pro-inflammatory cytokines such as IL-6 and TNF-α and IL-17, showing the presence of a general inflammation after allergen exposure. These cytokines may be produced by a variety of cells as structural cells i.e. epithelial or fibroblasts or immune cells such as mast cells or eosinophils. However, it is likely that T cells represent a major source of many of the measured cytokines, and that Th1 and Th2 cells produce their corresponding cytokines during proliferation [40].

We decided to take these findings further, to more exactly determine the local inflammatory milieu in the specific tissue microenvironment were respective T effector cells were found. Thus, laser microdissection and pressure catapulting technology and real-time RT-PCR analysis was combined to document the presence of expression of a broad range of inflammatory genes, mainly cytokines and chemokines, in respective tissue. Our data confirmed the hypothesis that there are differences local inflammatory milieus in the lung tissue compartments during allergic airway inflammation. Thus, from the 28 genes that had a fold changed ≥3 times after allergen exposure only 14, were common in all examined tissues, whereas some were dominating in respective environment. Importantly, even among these genes, differences in levels of expression were documented. For example, eotaxin-2 (Ccl24), which represents a major chemoattractant for eosinophils, showing a big variation in its local expression, can possibly explaining the results of high accumulation of eosinophils in perivascular tissue [10]. This chemokine may also have cytokine properties, as we have shown that it can play a role in eosinophilopoiesis locally in the lung [41]. Another example is the expression of osteopontin (Spp1), which recently has been proposed to enhance Th2 responses and is related to human asthma severity [42].

Chemokines plays a major role in T cell trafficking in allergic and asthmatic inflammation [43]. Therefore it was logical to hypothesise that local chemokine milieu can affect T effector cells accumulation. Indeed we have described extensive chemokine networks expressed in all investigated tissues, but with uneven expression in the different compartments. The pattern of their expression can at least partly explain our immunohistochemistry results. Thus, Th1 reported chemokines such as Ccl17 and Ccl2 [43], [44] were primarily expressed in alveolar tissues, where T-bet cells primarily are located. Ccl8 was quite recently reported by Islam *et al*. [45] as a potent chemoattractant for GATA-3, IL-5^+^ Th2 cells in skin allergy, which is in line with our finding of expression of this chemokine in primarily the perivascular milieu, where GATA-3 cells were accumulated. Further, the chemokines Ccl19 may explain the microenvironmental localisation of Treg, while the chemokines Ccl20 may not explain Th17 cells distribution [43], [46], [47]. By Ingenuity Pathways Analysis, we have documented extensive interactions between the chemokines and cytokines, suggesting that a small difference in expression of one may affect the action of the other, and can thus influence the whole inflammatory network in the microenvironment. This becomes clearer when some chemokines such as Ccl4 can influence the traffic of more than one of the studied T effector cells. The interaction between the different chemokines is complicated, since we observed a down-regulation of Pf4, which is related to Th1 responses [48], even though other Th1 chemokines were increased in the same environment.

In summary, our current study shows that T-bet, GATA-3, Foxp3 and RORγt in both CD25^+^ and CD25^−^ CD4^+^ T cells all expand in lung during allergic airway inflammation, and also proliferate within the lung. In this mouse model of allergic airway inflammation, Th2 cells are the most dominant effector T-cell. Within the lungs, these effector T cells are differently distributed, with Th1 primarily being present in alveolar lung tissue, Th2 and Treg cells primarily being observed in perivascular lung tissue, and the numbers of Th17 cells being few but with a dominance in peribronchial lung tissue. The inflammatory milieu in those tissue compartment showed different profiles, both for mediators associated to allergic inflammation, such as chemokines, explaining at least partly our results. Overall, this study shows that Th2 cells are prominent in allergic inflammation, but also verify concomitant the activity of Th1, Th2, Th17 and Tregs.
